# Supplementary material for: Predicting bloodstream infection outcome using machine learning
Source: Sci Rep. 2021 Oct 11;11:20101. doi: 10.1038/s41598-021-99105-2 (PMC8505419; doi:10.1038/s41598-021-99105-2)
Supplement: Supplementary file 2 — Supplementary Information 2. [file 41598_2021_99105_MOESM2_ESM.docx]

| - Bacillus cereus | - Streptococcus equi ssp equi |
| --- | --- |
| - Bacillus circulans | - Streptococcus equi ssp zooepidemicus |
| - Bacillus firmus | - Streptococcus gordonii |
| - Bacillus licheniformis | - Streptococcus intermedius |
| - Bacillus megaterium | - Streptococcus mitis |
| - Bacillus pumilus | - Streptococcus mitis/oralis |
| - Bacillus species | - Streptococcus mutans |
| - Bacillus sphaericus | - Streptococcus pyogenes |
| - Bacillus subtilis | - Streptococcus salivarius |
| - Coagulase negative Staphylococcus | - Streptococcus sanguinis |
| - Corynebacterium jeikeium | - Streptococcus vestibularis |
| - Corynebacterium species | - Streptococcus viridans group |
| - Corynebacterium xerosis | - Micrococcus luteus/lylae |
| - Micrococcus luteus | - Staphylococcus arlettae |
| - Micrococcus species | - Staphylococcus caprae |
| - Staphylococcus spp | - Staphylococcus carnosus ssp carnosus |
| - Staphylococcus aureus | - Staphylococcus gallinarum |
| - Staphylococcus auricularis | - Staphylococcus hominis ssp hominis |
| - Staphylococcus capitis | - Streptococcus alactolyticus |
| - Staphylococcus cohnii ssp cohnii | - Streptococcus cristatus |
| - Staphylococcus cohnii ssp urealyticus | - Streptococcus constellatus ssp constellatus |
| - Staphylococcus epidermidis | - Streptococcus constellatus ssp pharyngis |
| - Staphylococcus haemolyticus | - Streptococcus hyointestinalis |
| - Staphylococcus hominis | - Streptococcus mitis/Streptococcus oralis |
| - Staphylococcus intermedius | - Streptococcus parasanguinis |
| - Staphylococcus kloosii | - Streptococcus pluranimalium |
| - Staphylococcus lentus | - Streptococcus sobrinus |
| - Staphylococcus lugdunensis | - Streptococcus thermophilus |
| - Staphylococcus saccharolyticus | - Streptococcus thoraltensis |
| - Staphylococcus saprophyticus | - Streptococcus spp |
| - Staphylococcus schleiferi | - Diphtheroids spp |
| - Staphylococcus sciuri | - Corynebacterium striatum |
| - Staphylococcus simulans | - Non haemolytic streptococcis |
| - Staphylococcus species | - Babesia spp |
| - Staphylococcus warneri | - Corynebacterium minutissmum |
| - Staphylococcus xylosus | - Corynebacterium amycolatum |
| - Streptococcus agalactiae | - Micromonas micros |
| - Streptococcus anginosus | - Staphylococcus pasteuri |
| - Streptococcus constellatus |  |

**Supplementary file**: List of bacteria classified as contaminants
