# Supplementary material for: Predicting bloodstream infection outcome using machine learning
Source: Sci Rep. 2021 Oct 11;11:20101. doi: 10.1038/s41598-021-99105-2 (PMC8505419; doi:10.1038/s41598-021-99105-2)
Supplement: Supplementary file 3 — Supplementary Information 3. [file 41598_2021_99105_MOESM3_ESM.docx]

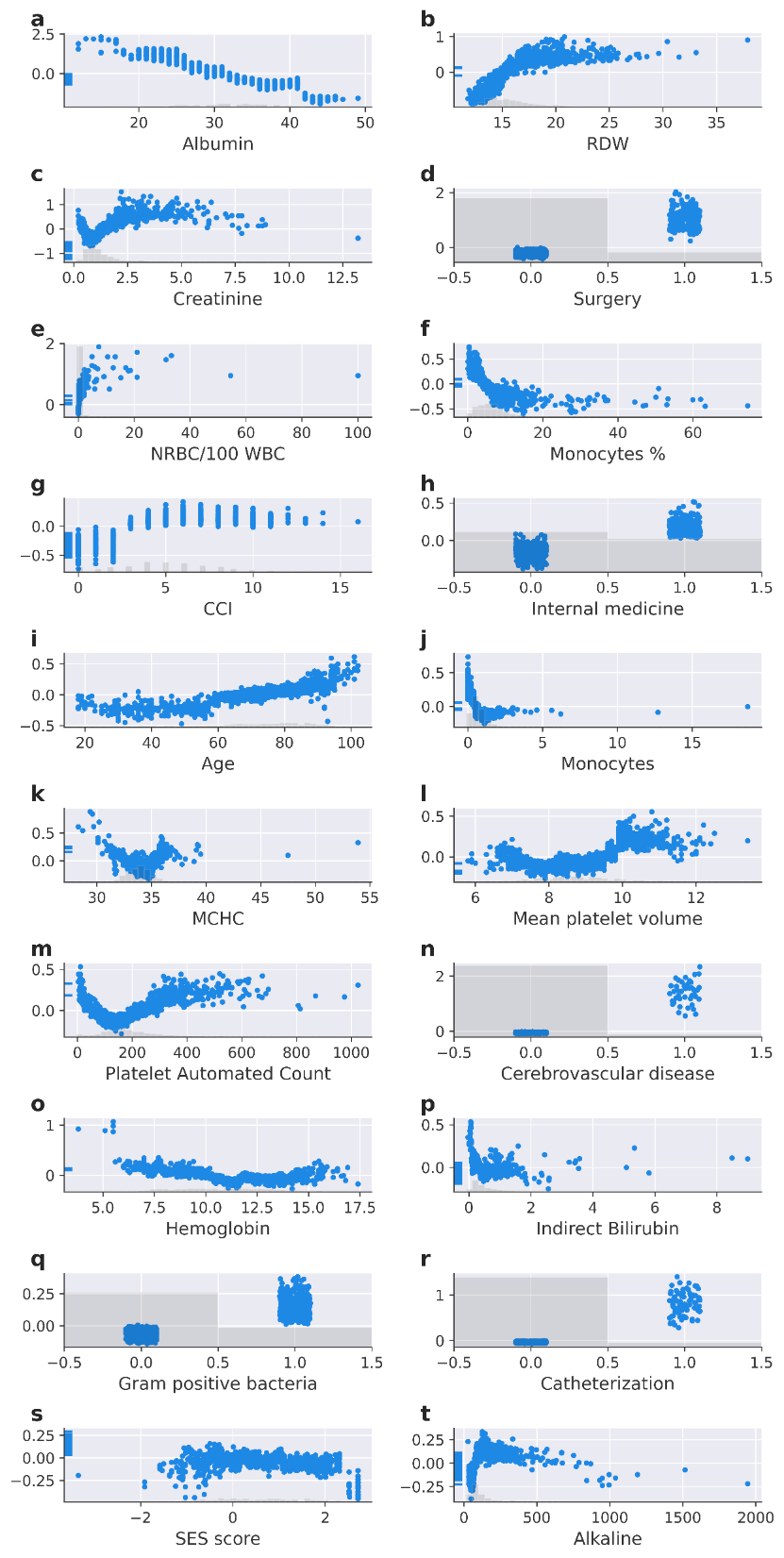


**Supplementary Figure 1. a-t.** Scatter plots of SHAP for the different values of the top 20 features for the inclusive model. The light histogram along the X-axis shows the density of the data. Albumin (g/L), RDW (%), Creatinine (mg/dL), NRBC/100 WBC (%), Monocytes (%), Age (years), Monocytes (10e3/ϻL), MCHC (g/dL), Mean platelet volume (fL), Platelet automated count (10e3/ϻL), Hemoglobin (g/dL), AST (U/L), indirect bilirubin (mg/dL), alkaline (U/L).


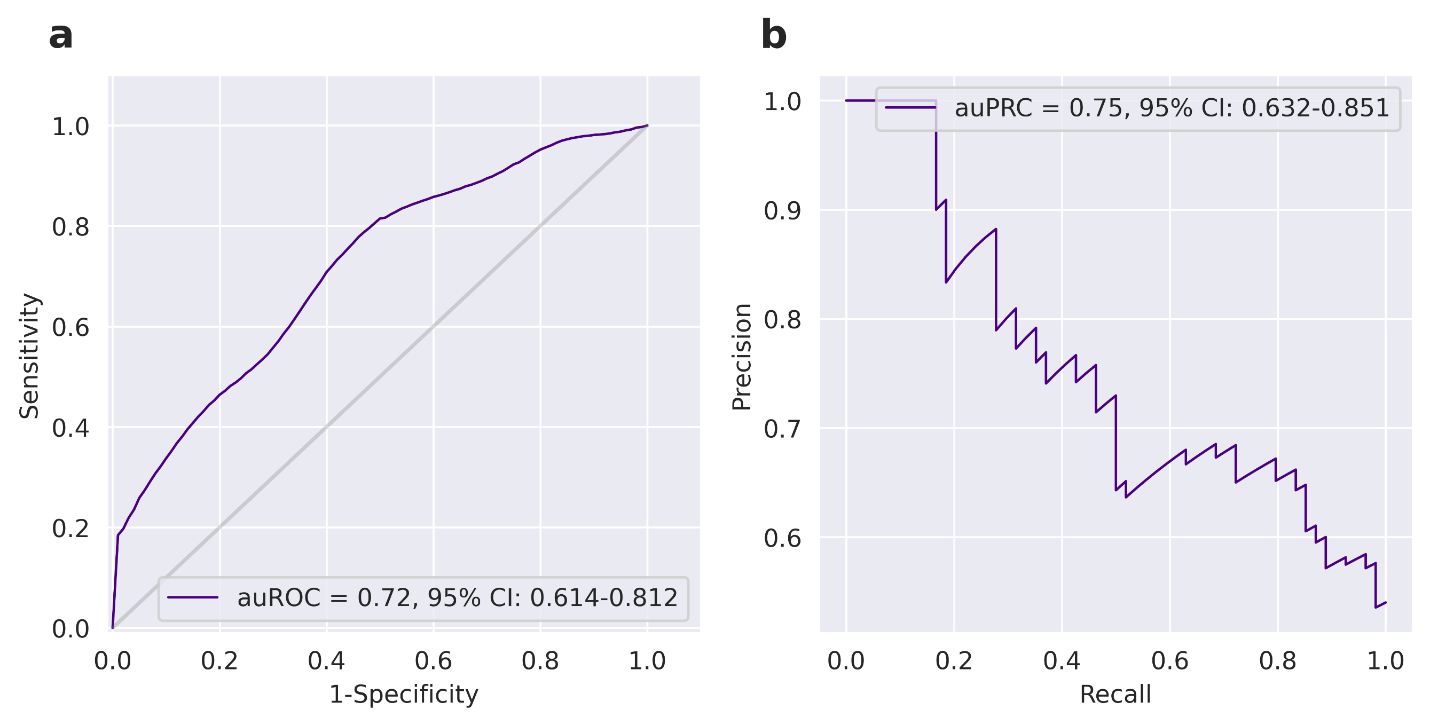


**Supplementary Figure 2. Performance of the inclusive model on ICU patients a**. Receiver-operating characteristics (ROC) curves. **b**. A plot of the precision (positive predictive value, PPV) against the recall (sensitivity) of the predictor for different thresholds.


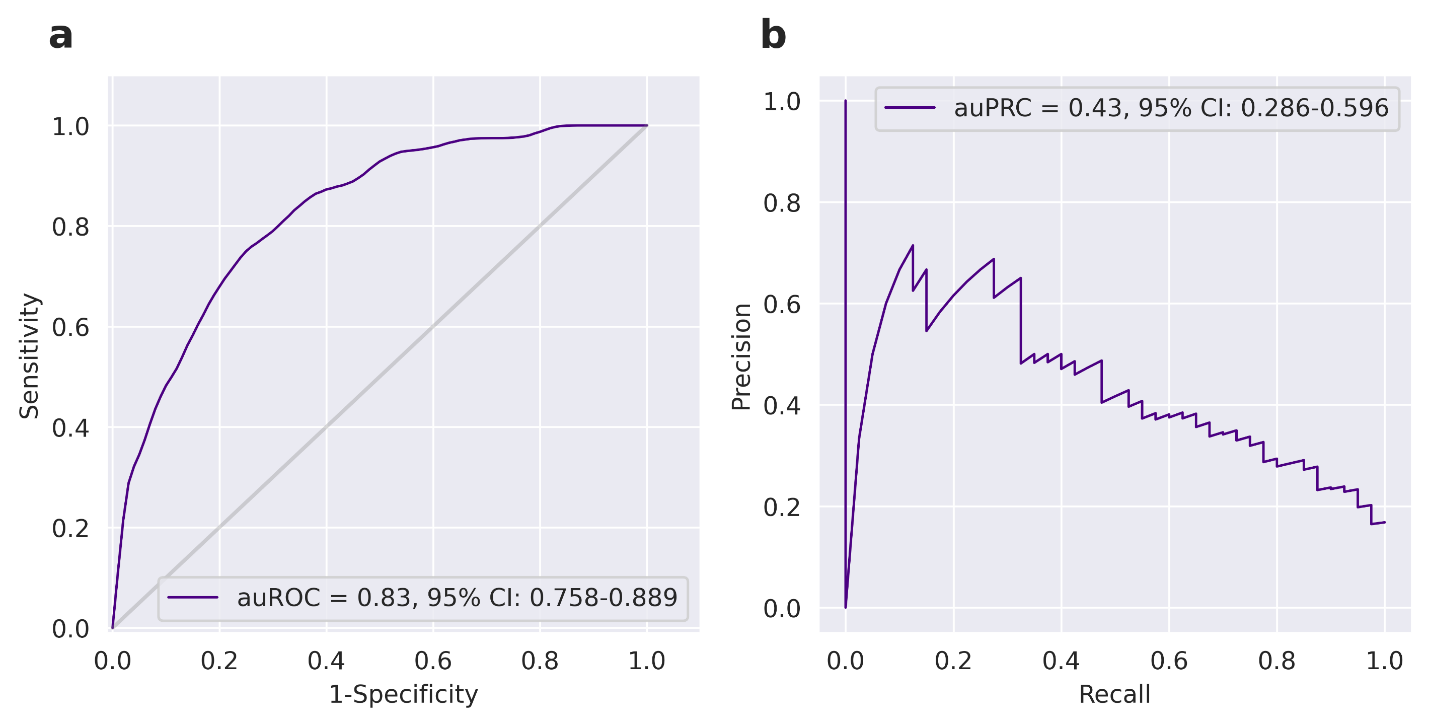


**Supplementary Figure 3. Performance of the inclusive model on ER patients a**. Receiver-operating characteristics (ROC) curves. **b**. A plot of the precision (positive predictive value, PPV) against the recall (sensitivity) of the predictor for different thresholds.


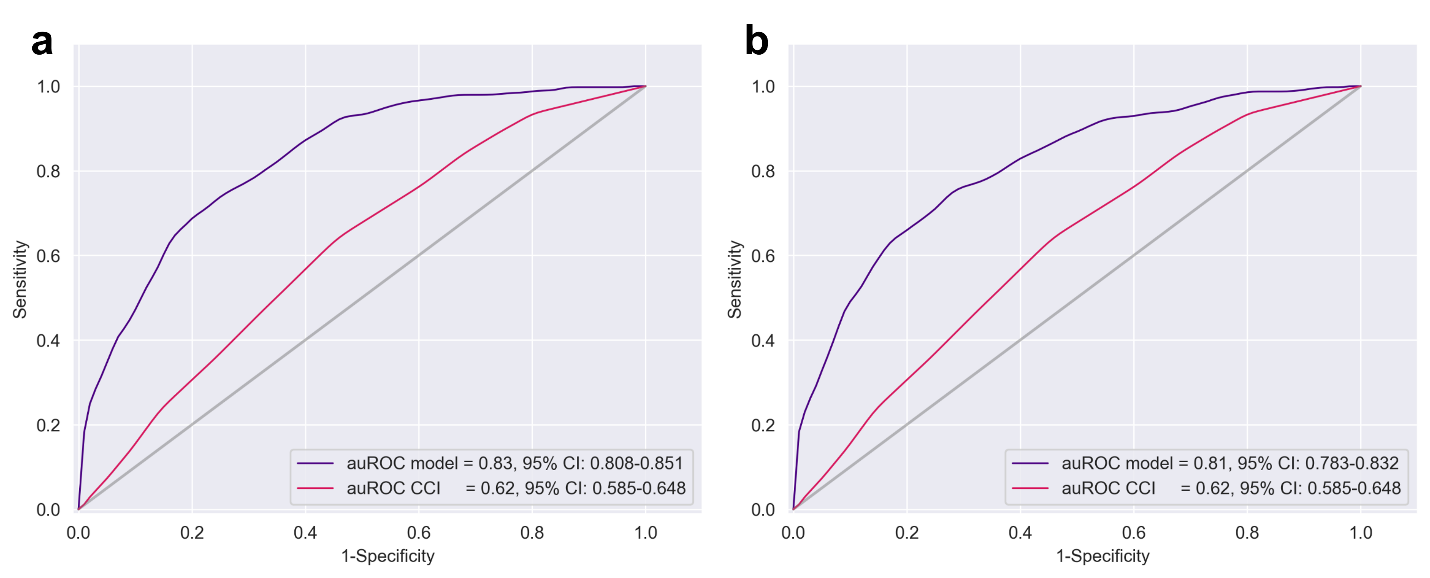


**Supplementary Figure 4. Comparison of CCI to our models a.** ROC curves of the inclusive model and CCI values (converted to probabilities) of the poor outcome**. b.** ROC curves of the compact model and CCI values (converted to probabilities) of the poor outcome.
